# Tumor Macroscopic Morphology Is an Important Prognostic Factor in Predicting Chemotherapeutic Efficacy and Clinical Outcomes of Patients With Colorectal Neuroendocrine Neoplasms, One Multicenter Retrospective Cohort Study

**DOI:** 10.3389/fendo.2021.801741

**Published:** 2021-12-20

**Authors:** Zhijie Wang, Ke An, Rui Li, Qian Liu

**Affiliations:** ^1^ Department of Colorectal Surgery, National Cancer Center/National Clinical Research Center for Cancer/Cancer Hospital, Chinese Academy of Medical Sciences and Peking Union Medical College, Beijing, China; ^2^ Department of General Surgery, China-Japan Friendship Hospital, Beijing, China; ^3^ Department of General Surgery, Beijing Hospital, Beijing, China

**Keywords:** colon, rectum, neuroendocrine tumors, morphology, endoscopy

## Abstract

**Background and Aims:**

Locally advanced and metastatic colorectal neuroendocrine neoplasm (NEN) is a rare disease with a dismal prognosis. We aimed to explore the value of the macroscopic morphology of NENs in the management of TNM stage II-IV colorectal NENs, which has not been fully elucidated in previous reports.

**Methods:**

We retrospectively enrolled 125 eligible patients with TNM stage II-IV colorectal NENs who were diagnosed between 2000 and 2020 from three Chinese hospitals. All were categorized into either protruding or ulcerative NEN groups through endoscopic evaluation of their macroscopic morphology. Clinicopathological data were collected and compared between the two groups. Survival analysis was performed to assess the survival outcomes between the two groups.

**Results:**

A total of 77 and 48 patients had protruding and ulcerative NENs, respectively. Patients with ulcerative NENs had a larger median tumor size (P<0.001) and higher median Ki-67 index (P<0.001), and a larger proportion of these patients had grade G3 disease (P=0.001) and poorly differentiated neoplasms (P=0.001), as well as higher frequencies of T3 and T4 tumors (P=0.006) than patients with protruding NENs. In addition, patients with ulcerative NENs showed a much lower response to first-line chemotherapy [50% (95% CI: 27.3% - 72.7%) versus 20% (95% CI: 3.1% - 36.9%), P=0.03] and a worse 3-year progression-free survival (PFS) rate [19.7% (95% CI: 7.2% - 32.2%) versus 49.5% (95% CI: 37.5% - 61.5%), P=0.001] and 3-year overall survival (OS) rate [30.7% (95% CI: 15.6% - 45.8%) versus 76.9% (95% CI: 66.5% - 87.3%), P<0.001] than those with protruding NENs. The multivariate analysis results indicated that the macroscopic shape of NENs was an independent prognostic factor affecting both PFS (HR = 1.760, 95% CI: 1.024 – 3.026, P = 0.04) and OS (HR = 2.280, 95% CI: 1.123 – 4.628, P = 0.02).

**Conclusions:**

Ulcerative NENs were more malignant and chemotherapy resistant than protruding NENs. Tumor macroscopic morphology is a valuable prognostic factor for stage II-IV colorectal NENs.

## Introduction

Colorectal neuroendocrine neoplasms (NENs) are derived from diffuse neuroendocrine cells throughout the colon and rectum (1). Although it is a rare disease, it has presented an increasing incidence in recent decades, owing to the popularization of colonoscopy screening (2–4). One study from the Netherlands indicated that the incidence of colorectal NENs doubled from 2006 to 2011, with incidence rates increasing from 0.36 per 100000 inhabitants to 0.75 per 100000 inhabitants (5).

Colorectal NENs are a group of heterogeneous diseases ranging from indolent tumors to highly aggressive carcinomas. In the World Health Organization (WHO) 2010 classification and nomenclature system for digestive NENs, colorectal NENs were classified into G1, G2 and G3 based on the mitotic count and/or Ki-67 index. G1 and G2 NENs were regarded as well-differentiated NENs, while G3 NENs were regarded as poorly differentiated neuroendocrine carcinomas (NECs) and included small cell carcinomas (SCCs) and large cell carcinomas (LCCs) (2). In the recent 2019 edition of the WHO classification system, well-differentiated G3 NENs are separated from NECs and termed G3 neuroendocrine tumors (NETs), which are less aggressive and present better clinical outcomes than NECs. G1 and G2 NENs and well-differentiated G3 NENs are collectively referred to as NETs (3, 6).

Most diagnosed colorectal NENs are small, indolent and localized lesions confined within the submucosal layer. One report based on the Surveillance, Epidemiology and End Results (SEER) database included 9602 cases with colorectal NENs, and localized NENs (Tis/T1N0M0) based on the European Neuroendocrine Tumor Society (ENETS) and Union for International Cancer Control/American Joint Committee on Cancer (UICC/AJCC) guidelines for TNM assessment of colorectal NENs accounted for 75.2% of all colorectal NENs (7). Therefore, most previous studies have focused on the management strategies for localized NENs and have indicated that endoscopic therapy is a reliable choice and could guarantee a favorable prognosis (8, 9). However, the optimal management scheme for locally advanced (T2-4N0M0 and T0-4N1M0) and metastatic (T0-4N0-1M1) NENs has not been well established due to its rarity and heterogeneity (10). Locally advanced and metastatic NENs refer to neoplasms invading into or through the muscularis propria or neoplasms with involvement of lymph nodes or distant metastasis, which are categorized as stage II-IV NENs based on the ENETS and UICC/AJCC guidelines (11). Although they constitute only a small proportion of diagnosed colorectal NENs, they present high malignancy and strong aggressiveness, which negatively affects the survival of patients.

Currently, the recognized prognostic factors include tumor size, grade, histological differentiation, depth of tumor invasion, status of regional lymph nodes and distant organ metastasis. The therapeutic scheme has been established based on comprehensive evaluation of these factors (2, 12). However, the tumor morphology has long been ignored in previous studies, even though it can be easily obtained through endoscopic examination. Although numerous prior reports have demonstrated the association between morphology and tumor characteristics for colorectal adenocarcinomas, there remain few studies on the value of morphology in the evaluation, treatment and surveillance of colorectal NENs (13, 14). NENs are typically divided into protruding and ulcerative lesions in patient medical records based on gross observation from colonoscopy examination. In this study, we aimed to determine whether the macroscopic morphological features of tumors had an impact on the clinical manifestations and outcomes of stage II-IV colorectal NENs.

## Materials And Methods

### Study Design

Our study received approval from the Ethics Committee of the National Cancer Center and complied with the Declaration of Helsinki of the World Medical Association. We performed a multicenter retrospective cohort study, and included 92 patients from Cancer Hospital Chinese Academy of Medical Sciences, 25 from China-Japan Friendship Hospital, and 8 from Beijing Hospital. Patients were categorized into a protruding subgroup and an ulcerative subgroup based on the endoscopic evaluation of tumor shape. Our primary outcomes of interest included tumor grade, depth of invasion, involvement of regional lymph nodes, distant organ metastasis and chemotherapeutic efficacy of first-line treatment. Secondary outcomes included cancer progression and disease-specific mortality.

### Tumor Morphology

**Figure 1:**
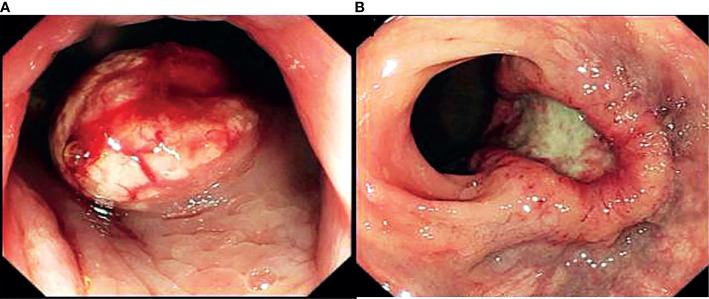
Endoscopic findings of macroscopic morphology of stage II-IV NENs. **(A)** protruding NENs, **(B)** ulcerative NENs.

Tumor shape was characterized based on endoscopic findings and was classified into protruding and ulcerative neoplasms. Lesions with obvious elevation over the surrounding normal mucosa were regarded as protruding tumors. Lesions with part of the mucosal surface that was lower than the surrounding normal mucosa were categorized into ulcerative tumors (**Figure 1**).

### Patients and Data

We retrospectively collected data from patients who received treatment at our institutions between 2000 and 2020. The inclusion criteria were as follows: 1) tumors located in the colon or rectum; 2) tumors pathologically confirmed as NENs; and 3) tumors that invaded into or through the muscularis propria, involved regional lymph nodes or showed distant metastasis. The exclusion criteria were as follows: 1) tumors located in the appendix; 2) tumors confined within the submucosa; 3) accompanying malignancies of other origin; and 4) a lack of complete data. From 2000 to 2020, 315 cases of colorectal NENs were diagnosed and treated at the National Cancer Center, Cancer Hospital Chinese Academy of Medical Sciences, China-Japan Friendship Hospital and Beijing Hospital. After excluding 145 patients with neoplasms confined to the submucosa, 33 patients with indeterminate tumor morphology, 7 patients with malignancies of other origins and 5 patients with NENs located in the appendix, 125 qualified patients with complete clinicopathologic and survival data were enrolled in our study. The data needed in our report were collected from either the hospital database or *via* telephone call. The last follow-up visit was July 1, 2021. Overall survival (OS) was calculated between the date of initial treatment and cancer-specific death. Progression-free survival (PFS) was obtained between the date of initial therapy and cancer progression based on imaging evaluations.

### Statistical Analysis

Continuous data that followed a normal distribution are presented as the mean ± standard deviations (SD) and were compared using a *t*-test. Continuous variables that did not follow the normal distribution are reported as median with interquartile range (IQR) and were analyzed using the Mann-Whitney *U* test. Categorical and ordinal factors are presented as frequency with percentage and were subsequently compared by *χ2* test for categorical data and Mann-Whitney *U* test for ordinal data. Cumulative incidence of cancer specific mortality (CSM) was calculated by a competing risk model, death from other causes was recognized as a competitive event of cancer-specific death. Gray’s test was used to determine the intergroup difference in the CSM. OS and PFS rates were determined using the Kaplan–Meier method. Univariable and multivariable logistic regression analyses were utilized to determine the relationship between macroscopic morphological patterns and prognosis. All data were calculated and analyzed using the Statistical Package for the Social Sciences (SPSS version 24.0; IBM Corp., Armonk, NY). Statistical significance was set at a two-sided P-value<0.05.

## Results

### Patient Characteristics

Patient demographics and clinicopathological manifestations are summarized in **Table 1**. A total of 125 patients with a mean age of 56.1 ± 11.8 years old and mean body mass index (BMI) of 24.3 ± 3.0 kg/m^2^ were enrolled in our study, consisting of 81 (64.8%) male and 44 (35.2%) female patients. Most patients (75.2%) had NENs in the rectum, followed by the cecum and ascending colon (9.6%), sigmoid colon (8.8%), descending colon (4.0%) and transverse colon (2.4%). All NENs had a median size of 3.0 (IQR 2.0–5.0) cm, with 16.8%, 22.4% and 60.8% having G1, G2 and G3 grades, respectively. Sixty-four patients (51.2%) were pathologically confirmed to have poorly differentiated disease and were categorized into the NEC group, and the remaining 61 patients (48.8%) were found to have well-differentiated disease and categorized into the NET group. In terms of the immunohistochemical markers, the expression of synaptophysin, chromogranin and CD 56 was detected in 94.4%, 67.8% and 87.5% of evaluable patients, respectively. The median Ki-67 index in the whole cohort was 40.0% (IQR 5.0%-70.0%). Most patients with NENs had tumors invading through the muscularis propria (68.8%) and involving regional lymph nodes (84.0%). Forty-eight (38.4%) patients had distant metastasis at the initial date of diagnosis. Based on the ENETS and UICC/AJCC TNM assessment of colorectal NENs, 15 (12.0%), 62 (49.6%) and 48 (38.4%) patients were classified as having stage II, III and IV disease, respectively. Extramural vascular invasion (EMVI) and perineural invasion (PNI) were found in 54.3% and 43.6% of the evaluable patients, respectively. With regard to the treatment regimens, surgery, chemotherapy and radiotherapy were performed in 80.8%, 75.2% and 16.8% of the patients, respectively.

**Table 1:** Demographic and clinicopathological manifestations.

Variables	All (n = 125)	Protruding (n = 77)	Ulcerative (n = 48)	P-value
Gender, n (%)				0.97
Male	81 (64.8%)	50 (64.9%)	31 (64.6%)	
Female	44 (35.2%)	27 (35.1%)	17 (35.4%)	
Age (yr, mean ± SD)	56.1 ± 11.8	54.9 ± 11.0	58.0 ± 12.7	0.15
BMI (kg/m^2^, mean ± SD)	24.3 ± 3.0	24.6 ± 3.4	23.9 ± 3.0	0.29
Family history of cancer, n (%)	28 (22.4%)	19 (25.0%)	9 (18.8%)	0.45
Smoking	44 (35.2%)	24 (31.2%)	20 (41.7%)	0.22
Alcohol consumption	43 (34.4%)	27 (35.1%)	16 (33.3%)	0.93
Location, n (%)				0.67
Rectum	94 (75.2%)	61 (79.2%)	33 (68.8%)	
Sigmoid colon	11 (8.8%)	6 (7.8%)	5 (10.4%)	
Descending colon	5 (4.0%)	2 (2.6%)	3 (6.3%)	
Transverse colon	3 (2.4%)	2 (2.6%)	1 (2.1%)	
Cecum and ascending colon	12 (9.6%)	6 (7.8%)	6 (12.5%)	
Size (cm), median (IQR)	3.0 (2.0, 5.0)	2.5 (1.7, 4.0)	5.0 (3.0, 7.0)	<0.001
Grade				0.001
G1	21 (16.8%)	18 (23.4%)	3 (6.25%)	
G2	28 (22.4%)	22 (28.6%)	6 (12.5%)	
G3	76 (60.8%)	37 (48.1%)	39 (81.3%)	
Differentiation				0.001
NET	61 (48.8%)	47 (61.0%)	14 (29.2%)	
NEC	64 (51.2%)	30 (39.0%)	34 (70.8%)	
Synaptophysin, n (%)	118 (94.4%)	72 (93.5%)	46 (95.8%)	>0.99
Chromogranin, n (%)	78 (67.8%)	46 (59.7%)	32 (66.7%)	0.53
CD56, n (%)				0.27
Positive	91 (72.8%)	63 (90.0%)	28 (82.4%)	
Negative	13 (10.4%)	7 (10.0%)	6 (17.6%)	
Unknown	21 (16.8%)	7 (9.1%)	14 (29.2%)	
Ki-67 (%), median (IQR)	40.0% (5.0%, 70.0%)	10.0% (3.0%, 60.0%)	60.0% (27.5%, 80.0%)	<0.001
T stage, n (%)				0.006
T1, T2	39 (31.2%)	31 (40.3%)	8 (16.7%)	
T3, T4	86 (68.8%)	46 (59.7%)	40 (83.3%)	
N stage, n (%)				0.73
N0	20 (16.0%)	13 (16.9%)	7 (14.6%)	
N1	105 (84.0%)	64 (83.1%)	41 (85.4%)	
M stage, n (%)				0.18
M0	77 (61.6%)	51 (66.2%)	26 (54.2%)	
M1	48 (38.4%)	26 (33.8%)	22 (45.8%)	
TNM stage, n (%)				0.40
II	15 (12.0%)	10 (13.0%)	5 (10.4%)	
III	62 (49.6%)	41 (53.2%)	21 (43.8%)	
IV	48 (38.4%)	26 (33.8%)	22 (45.8%)	
EMVI, n (%)				0.11
Positive	51 (40.8%)	30 (48.4%)	21 (65.6%)	
Negative	43 (34.4%)	32 (51.6%)	11 (34.4%)	
Unknown	31 (24.8%)	15 (19.5%)	16 (33.3%)	
PNI, n (%)				0.67
Positive	41 (32.8%)	28 (45.2%)	13 (40.6%)	
Negative	53 (42.4%)	34 (54.8%)	19 (59.4%)	
Unknown	31 (24.8%)	15 (19.5%)	16 (33.3%)	
Surgery	101 (80.8%)	66 (85.7%)	35 (72.9%)	0.08
Chemotherapy	94 (75.2%)	56 (72.7%)	38 (79.2%)	0.42
Radiotherapy	21 (16.8%)	10 (13.0%)	11 (22.9%)	0.15

Patients with unknown information were not included in the χ^2^ -test.

SD, standard deviation; BMI, body mass index; IQR, interquartile range; NET, neuroendocrine tumor; NEC, neuroendocrine carcinoma; EMVI, extramural vascular invasion; PNI, perineural invasion.

In the whole cohort, 77 (61.6%) and 48 (38.4%) patients were characterized as having protruding and ulcerative lesions, respectively. Statistical analysis showed that there were no significant discrepancies between the groups in terms of the distribution of sex (P= 0.97), age (P=0.15), BMI (P=0.29), family history of cancer (P=0.45), smoking (P=0.22), alcohol consumption (P=0.93), location (P=0.67), positive rates of synaptophysin (P>0.99), chromogranin (P=0.53) and CD 56 (P=0.27), regional lymph node status (P=0.73), distant metastasis (P=0.18), TNM stage (P=0.40), EMVI (P=0.11), PNI (P=0.67), and intervention by surgery (P=0.08), chemotherapy (P=0.42), or radiotherapy (P=0.15). The Mann-Whitney *U* test demonstrated that the patients with ulcerative NENs presented a larger median size (5.0 cm in the ulcerative group versus 2.5 cm in the protruding group, P<0.001) and higher median Ki-67 index (60.0% in the ulcerative group versus 10.0% in the protruding group, P<0.001) than patients with protruding NENs. With regard to the grade and differentiation of NENs, a higher proportion of patients with ulcerative NENs had grade G3 disease (81.3% in the ulcerative group versus 48.1% in the protruding group, P=0.001) and poorly differentiated NEC neoplasms (70.8% in the ulcerative group versus 30.9% in the protruding group, P=0.001). In terms of the depth of cancer invasion, the patients with ulcerative NENs were more prone to experiencing invasion through the muscularis propria; 40 (83.3%) and 46 (59.7%) in the ulcerative group and protruding group presented T3 and T4 stage tumors, respectively (P=0.006).

### The Predictive Value of Morphology for NEN Patients Receiving First-Line Chemotherapy

Detailed information regarding the first-line chemotherapy schedule and treatment efficacy was available for 47 patients, including 35 patients who had distant metastasis at the initial diagnosis and 12 patients who had local NENs but experienced progression after radical surgical treatment. Of the 47 patients, 16 responded to first-line chemotherapy, with an overall response rate of 34%. Eleven of the 22 patients with protruding NENs and 5 of the 25 patients with ulcerative NENs responded to first-line chemotherapy, with response rates of 50.0% and 25.0%, respectively (**Table 2**). Patients with ulcerative NENs were significantly less sensitive to chemotherapy (P=0.03).

**Table 2:** Data regarding NENs received first-line chemotherapy.

	Chemotherapy regimens	Patients received first-line chemotherapy	Patients responded to first-line chemotherapy	In total	P-value
Protruding (n=22)	cisplatin/carboplatin+etoposide	10	7	11 (50.0%)	0.03^*^
	oxaliplatin+capecitabine/5-Fu	5	1		
	temozolomide + capecitabine	1	0		
	temozolomide + S-1	3	2		
	irinotecan + S-1	1	1		
	etoposide + thalidomide	1	0		
	AK105 + anlotinib	1	0		
Ulcerative (n=25)	cisplatin/carboplatin/oxaliplatin+etoposide	11	3	5 (20.0%)	
	oxaliplatin+capecitabine/5-Fu	7	1		
	temozolomide + capecitabine	3	0		
	irinotecan + capecitabine	1	1		
	cisplatin + irinotecan	1	0		
	oxaliplatin +fruquintinib	1	0		
	AK105 + anlotinib	1	0		

*The P-value refers to the comparison between response rates in protruding and ulcerative groups.

### Oncological Outcomes

**Figure 2:**
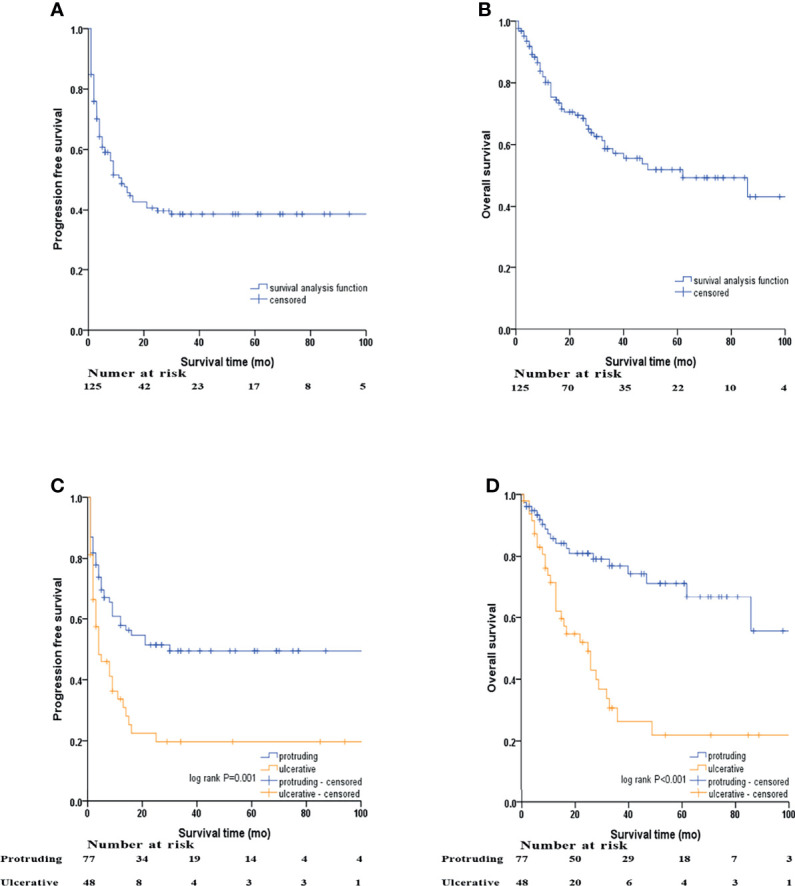
Kaplan–Meier survival analyses by macroscopic morphology of NENs. **(A)** PFS of the whole cohort, **(B)** OS of the whole cohort, **(C)** PFS of protruding and ulcerative NENs in the whole cohort, **(D)** OS of the protruding and ulcerative NENs in the whole cohort.

A median follow-up period of 26 months (range 1–183 months) was reached in our research. Eight patients were lost to follow-up due to loss of communication or unexpected death from other accidents, resulting in a follow-up completion rate of 93.6%. Kaplan-Meier survival curves were used to determine the PFS and OS rates of the whole cohort and for subgroup analyses by NEN morphology (**Figure 2**).

In the whole cohort, the 3-year PFS and OS rates were 38.4% (95% CI: 29.2% - 47.6%) and 57.2% (95% CI: 47.2% - 67.2%), respectively, with the median PFS and OS being 12 and 62 months, respectively. We subsequently evaluated the difference in survival outcomes between protruding and ulcerative NENs. In the protruding group, the 3-year PFS rate was 49.5% (95% CI: 37.5% - 61.5%), and the median PFS was 30 months. In the ulcerative group, the 3-year PFS rate was 19.7% (95% CI: 7.2% - 32.2%), and the median PFS was only 4 months. In terms of OS, the 3-year OS rates were 76.9% (95% CI: 66.5% - 87.3%) and 30.7% (95% CI: 15.6% - 45.8%) for the protruding and ulcerative groups, respectively. The median OS in the ulcerative group was 25 months, while the median OS in the protruding group could not be calculated, as more than half of the patients were still alive by the end of our follow-up. Patients with ulcerative NENs had significantly worse PFS (log-rank P=0.001) and OS (log-rank P<0.001) rates than those with protruding NENs.

### Stratification Analysis Based on the Presence or Absence of Distant Metastasis

Tumor features stratified by TNM stage and morphology are shown in **Table 3**. Given the limited sample size of our study, patients with TNM stage II and III disease were analyzed together as regional disease. For individuals with regional NENs, we still found that patients with ulcerative lesions were prone to neoplasms of a larger size (P=0.002), higher grade (P=0.003), and poorer histological differentiation (P=0.008) with a higher Ki-67 index (P=0.02) and deeper layers of intestinal wall invasion (P=0.03) than patients with protruding lesions. In terms of patients with metastatic disease, only size (P=0.001), tumor grade (P=0.04) and histological differentiation (P=0.03) demonstrated a significant difference between patients with protruding and ulcerative NENs. No obvious discrepancies in the Ki-67 index (P=0.13) or T stage (P=0.45) were observed between the two groups.

**Table 3:** Stratification analysis by presence or absence of distant metastasis.

Variables	Regional NENs			Metastatic NENs		
	Protruding (n = 51)	Ulcerative (n = 26)	P-value	Protruding (n = 26)	Ulcerative (n = 22)	P-value
Size (cm), median (IQR)	2.1 (1.5, 3.6)	4.0 (2.0, 7.5)	0.002	3.0 (2.1, 4.0)	5.0 (4.0, 6.0)	0.001
Grade, n (%)			0.003			0.04
G1 and G2	28 (54.9%)	5 (19.2%)		12 (46.2%)	4 (18.2%)	
G3	23 (45.1%)	21 (80.8%)		14 (53.8%)	18 (81.8%)	
Differentiation, n (%)			0.008			0.03
NET	32 (62.7%)	8 (30.8%)		15 (57.7%)	6 (27.3%)	
NEC	19 (37.3%)	18 (69.2%)		11 (42.3%)	16 (72.7%)	
Ki-67 (%), median (IQR)	5.0% (2.0%, 50.0%)	65.0% (20.0%, 80.0%)	0.02	25.0% (5.0%, 65.0%)	60.0% (29.0%, 80.0%)	0.13
T stage, n (%)			0.03			0.45
T1, T2	27 (52.9%)	7 (26.9%)		4 (15.4%)	1 (4.5%)	
T3, T4	24 (47.1%)	19 (73.1%)		22 (84.6%)	21 (95.5%)	

IQR, interquartile range; NET, neuroendocrine tumor; NEC, neuroendocrine carcinoma; NEN, neuroendocrine neoplasm.

**Figure 3:**
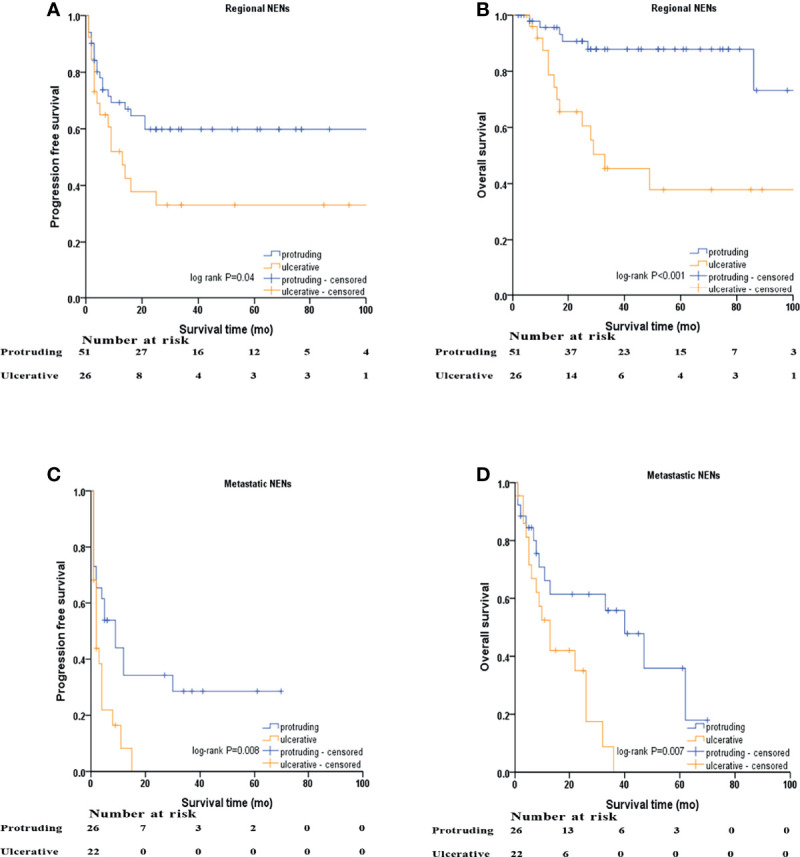
Kaplan–Meier survival analyses after stratified by M stage and macroscopic morphology of NENs. **(A)** PFS of protruding and ulcerative NENs in the patients with regional NENs, **(B)** OS of protruding and ulcerative NENs in the patients with regional NENs, **(C)** PFS of protruding and ulcerative NENs in the patients with metastatic NENs, **(D)** OS of protruding and ulcerative NENs in the patients with metastatic NENs.

Kaplan–Meier survival curves based on stratification analysis of TNM stages and tumor morphology were also performed (**Figure 3**). For patients with regional NENs, ulcerative NENs were significantly associated with decreased 3-year PFS [33.1% (95% CI: 13.7% - 52.5%) in ulcerative NENs versus 59.8% (95% CI: 45.7% - 73.9%) in protruding cases, log-rank P=0.04] and OS [45.4% (95% CI: 24.2% - 66.6%) in ulcerative NENs versus 87.9% (95% CI: 77.9% - 97.9%) in protruding cases, log-rank P<0.001]. For patients with metastatic NENs, ulcerative NENs were also associated with a worse 3-year PFS [0 in ulcerative NENs versus 28.6% (95% CI: 9.4% - 47.8%) in protruding cases, log-rank P=0.008] and OS [0 in ulcerative NENs versus 55.8% (95% CI: 34.8% - 76.8%) in protruding cases, log-rank P=0.007].

### Stratification Analysis Based on Tumor Size

Tumor features stratified by tumor size and morphology are shown in **Table 4**. For NENs ≤ 2.0 cm, ulcerative lesions were characterized with higher grade (P=0.007), and poorer histological differentiation (P=0.001) with a higher Ki-67 index (P=0.001) and deeper layers of intestinal wall invasion (P=0.02) than protruding lesions. Regarding NENs > 2.0 cm, ulcerative group showed a higher proportion of NENs with G3 grade, poor histological differentiation and higher Ki-67 index than protruding group. However, this tendency did not reach statistical significance, which might be due to the limited sample size of our study.

**Table 4:** Stratification analysis by tumor size.

Variables	Size ≤ 2cm			Size > 2cm		
	Protruding (n = 31)	Ulcerative (n = 10)	P-value	Protruding (n = 46)	Ulcerative (n = 38)	P-value
Grade, n (%)			0.007			0.06
G1 and G2	23 (74.2%)	2 (20.0%)		17 (37.0%)	7 (18.4%)	
G3	8 (25.8%)	8 (80.0%)		29 (63.0%)	31 (81.6%)	
Differentiation, n (%)			0.001			0.19
NET	26 (83.9%)	2 (20.0%)		21 (45.7%)	12 (31.6%)	
NEC	5 (16.1%)	8 (80.0%)		25 (54.3%)	26 (68.4%)	
Ki-67 [median (IQR)]	4.0% (2.0%, 10.0%)	70.0% (35.0%, 80.0%)	0.001	40.0% (5.0%, 62.5%)	60.0% (23.8%, 80.0%)	0.09
T stage, n (%)			0.02			0.79
T1, T2	24 (52.9%)	3 (30.0%)		7 (15.2%)	5 (13.2%)	
T3, T4	7 (47.1%)	7 (70.0%)		39 (84.8%)	33 (86.8%)	

NET, neuroendocrine tumor; NEC, neuroendocrine carcinoma.

**Figure 4:**
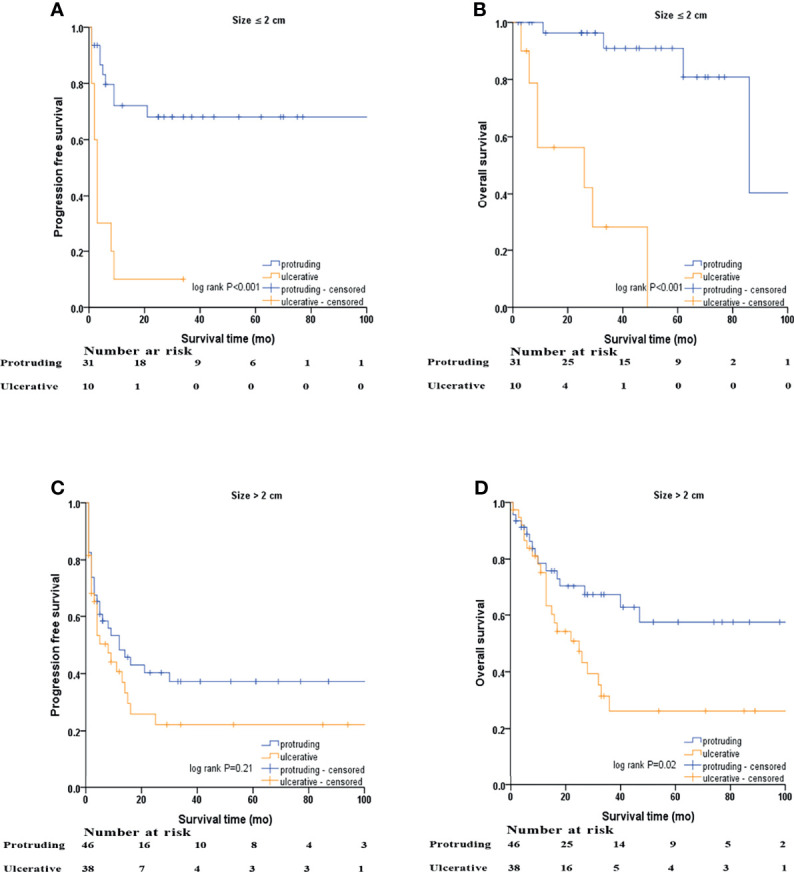
Kaplan–Meier survival analyses after stratified by tumor size and macroscopic morphology of NENs. **(A)** PFS of protruding and ulcerative NENs in the patients with NENs below 2 cm, **(B)** OS of protruding and ulcerative NENs in the patients with NENs below 2 cm, **(C)** PFS of protruding and ulcerative NENs in the patients with NENs above 2 cm, **(D)** OS of protruding and ulcerative NENs in the patients with NENs above 2 cm.

Kaplan–Meier survival curves based on stratification analysis of tumor size and morphology are presented (**Figure 4**). For patients with NENs ≤ 2.0 cm, ulcerative NENs were significantly associated with decreased 3-year PFS [10.0% (95% CI: 0 - 28.6%) in ulcerative NENs versus 68.1% (95% CI: 50.7% - 85.5%) in protruding cases, log-rank P<0.001] and OS [28.1% (95% CI: 0 – 60.0%) in ulcerative NENs versus 90.9% (95% CI: 78.7% - 100%) in protruding cases, log-rank P<0.001]. For patients with NENs > 2.0 cm, the 3-year PFS rates were 22.2% (95% CI: 7.3% - 37.1%) and 37.2% (95% CI: 22.3% - 52.1%) in the ulcerative and protruding NENs, respectively, which was not statistically different (log-rank P=0.21). However, we still observed significant decreased 3-year OS rate in ulcerative patients compared to protruding patients [26.2% (95% CI: 9.1% - 43.3%) in ulcerative NENs versus 67.2% (95% CI: 52.3% - 82.1%) in protruding cases, log-rank P=0.02].

### Cumulative Incidence of Death and Competing Risk Analysis

**Figure 5:**
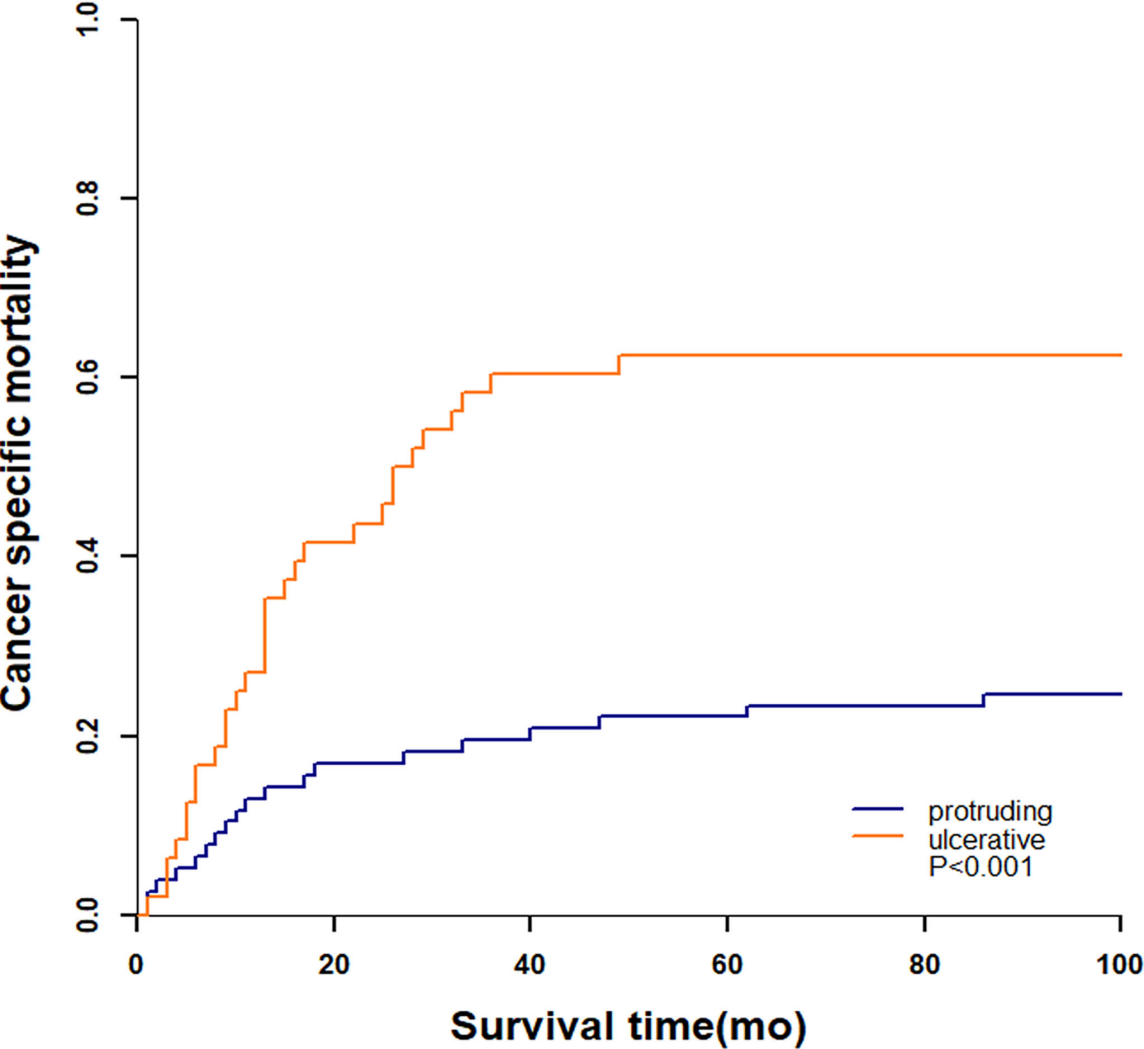
Cancer specific mortality of patients from competing risk model.

A total of 51 (40.8%) patients died by the end of our follow-up, of which 49 (96.1%) died from colorectal NENs and 2 (3.9%) died from other diseases. The 3-year cumulative incidence of NENs-specific death were 60.4% and 19.5% in ulcerative group and protruding group, respectively (**Figure 5**). Patients with ulcerative NENs had significantly increased CSM (P<0.001).

### Univariate and Multivariate Cox Regression Analyses

Univariable Cox proportional hazards regression analysis was performed to identify variables showing a significant association with DFS and OS, and the identified factors were enrolled in subsequent multivariable Cox proportional hazards regression analysis to evaluate the value of tumor morphology in predicting prognosis (**Tables 5**, **6**). After controlling for confounding factors, an ulcerative growth pattern (HR=1.760; 95% CI=1.024–3.026; P=0.04) and M1 stage (HR=2.006; 95% CI=1.067–3.774; P=0.03) were confirmed to be an independent risk factor associated with cancer progression. After controlling for confounding factors, ulcerative NENs (HR=2.280; 95% CI=1.123–4.628; P=0.02), age > 60 (HR=2.055; 95% CI=1.025–4.120; P=0.04), poor histological differentiation (HR=4.713; 95% CI=1.345–16.516; P=0.02) and M1 stage (HR=3.651; 95% CI=1.601–8.327; P=0.002) were significantly associated with increased mortality.

**Table 5:** Univariable and multivariable Cox proportional hazards regression analysis for PFS.

Variables	Univariable			Multivariable		
	HR	95% CI	P-value	HR	95% CI	P-value
Morphology (ulcerative vs protruding)	2.121	1.325-3.393	0.002	1.760	1.024-3.026	0.04
Size (>2 cm vs ≤ 2cm)	2.027	1.120-3.667	0.02	.880	0.385-2.012	0.76
Differentiation (NEC vs NET)	2.308	1.409-3.780	0.001	1.724	0.957-3.105	0.07
T stage (T3 T4 vs T1, T2)	2.599	1.422-4.749	0.002	1.421	0.577-3.498	0.45
M stage (M1 vs M0)	2.632	1.639-4.225	<0.001	2.006	1.067-3.774	0.03
Surgery (yes vs no)	0.398	0.233-0.680	0.001	0.693	0.348-1.381	0.30

PFS, progression free survival; NET, neuroendocrine tumor; NEC, neuroendocrine carcinoma; HR, hazards ratio; CI, confidence interval.

**Table 6:** Univariable and multivariable Cox proportional hazards regression analysis for OS.

Variables	Univariable			Multivariate		
	HR	95% CI	P-value	HR	95% CI	P-value
Age (> 60 vs ≤ 60)	2.515	1.424-4.443	0.001	2.055	1.025-4.120	0.04
Morphology (ulcerative vs protruding)	3.428	1.915-6.137	<0.001	2.280	1.123-4.628	0.02
Location (colon vs rectum)	1.914	1.051-3.486	0.03	0.922	0.453-1.877	0.82
Size (> 2cm vs ≤2cm)	2.673	1.242-5.755	0.01	1.041	0.322-3.368	0.95
Grade (G3 vs G1, G2)	3.576	1.674-7.639	0.001	0.904	0.222-3.686	0.89
Differentiation (NEC vs NET)	4.401	2.240-8.645	<0.001	4.713	1.345-16.516	0.02
T stage (T3, T4 vs T1, T2)	3.146	1.412-7.008	0.005	0.658	0.184-2.347	0.52
N stage (N1 vs N0)	3.634	1.125-11.742	0.03	2.458	0.699-8.644	0.16
M stage (M1 vs M0)	4.313	2.382-7.808	<0.001	3.651	1.601-8.327	0.002
Surgery (yes vs no)	0.313	0.166-0.591	<0.001	0.582	0.240-1.414	0.23

OS ,overall survival; NET, neuroendocrine tumor; NEC, neuroendocrine carcinoma; HR, hazards ratio; CI, confidence interval.

### Subgroup Analyses

**Figure 6:**
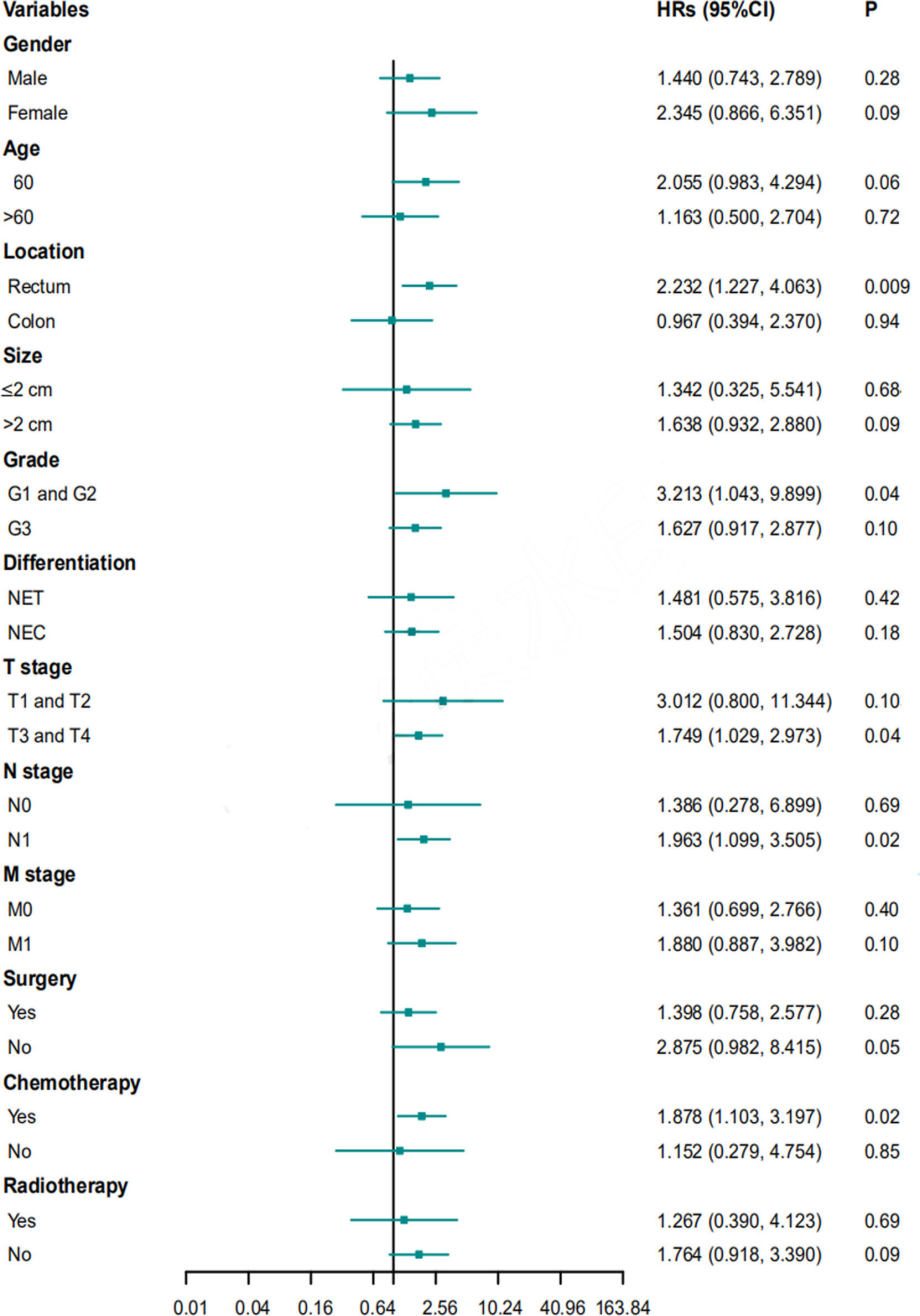
HRs with 95% CIs for PFS comparing protruding NENs and ulcerative NENs in different subgroups. HRs, hazard ratios; CI, confidence interval; NET, neuroendocrine tumor; NEC, neuroendocrine carcinoma; PFS, progression free survival.

**Figure 7:**
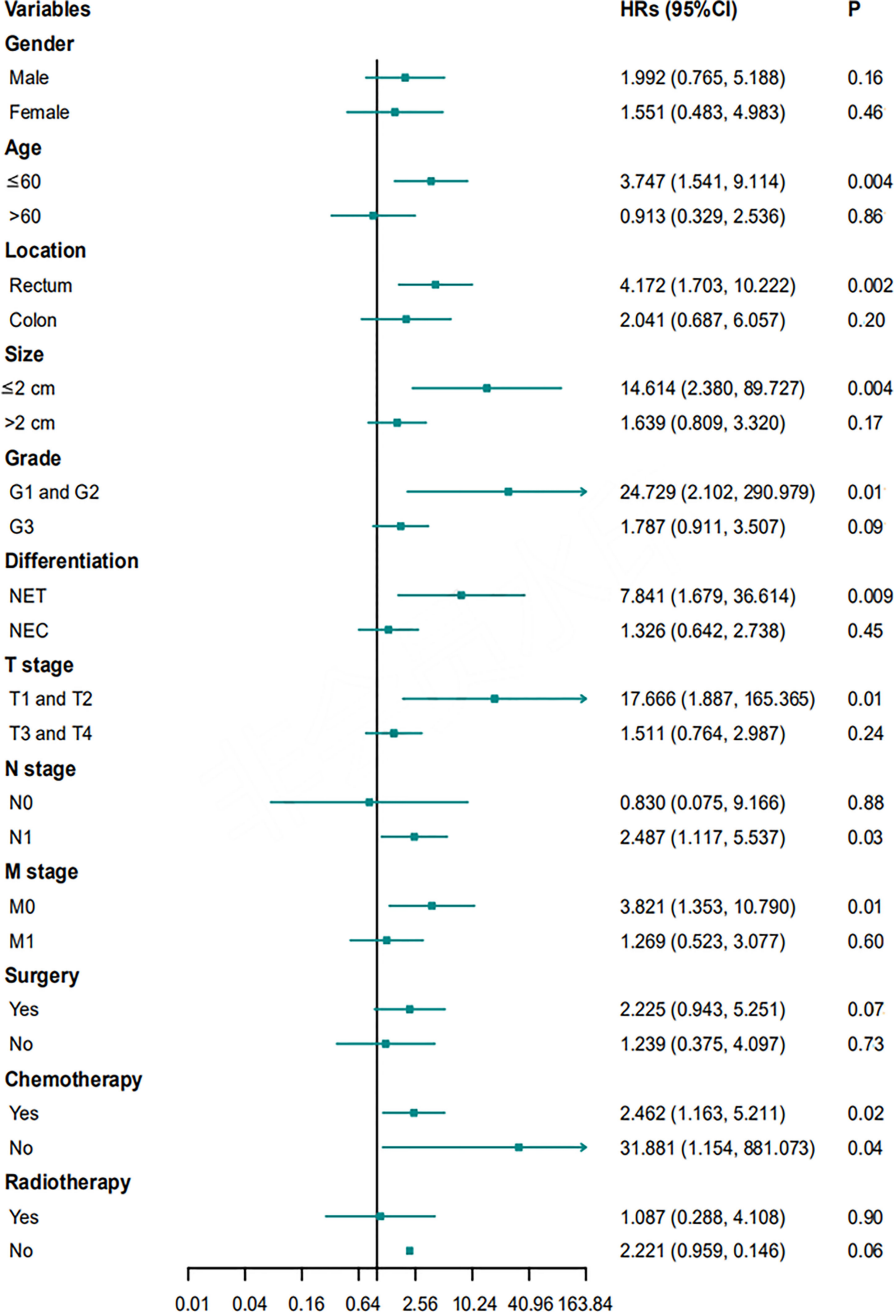
HRs with 95% CIs for OS comparing protruding NENs and ulcerative NENs in different subgroups. HRs, hazard ratios; CI, confidence interval; NET, neuroendocrine tumor; NEC, neuroendocrine carcinoma; OS, overall survival.

Multivariate Cox proportional hazard regression analyses for PFS and OS were performed for subgroups based on sex, age, location, size, grade, differentiation, T stage, N stage, M stage, surgery, chemotherapy, or radiotherapy. Ulcerative NENs were associated with worse PFS in subgroups of patients with rectal NENs, patients with G1 and G2 NENs, patients with T3 and T4 stage NENs, patients with N1 stage NENs, and patients who received chemotherapy (**Figure 6**). In terms of OS, patients with ulcerative NENs had poorer OS than those with protruding NENs in subgroups with a younger age, rectal NENs, smaller lesions, G1 and G2 grade NENs, NETs, T1 and T2 stage, N1 stage, and M0 stage as well as patients who received chemotherapy and those who did not (**Figure 7**).

## Discussion

Colorectal NENs are highly heterogeneous tumors with significantly different clinicopathological features and clinical outcomes. Endoscopic examination is essential, as the endoscopic appearance of NENs can provide an indispensable reference for subsequent diagnosis and treatment regimens. In prior reports, NEN size measured by endoscopy has been widely acknowledged as an important prognostic factor, and a NEN size of 2 cm was set as the cutoff point for endoscopic or radical surgical treatment of Tis/T1N0M0 NENs (11, 15, 16). However, even for diminutive NENs below 10 mm, lymph node metastasis can be observed and lead to a dismal prognosis (17, 18). Therefore, size alone is not a reliable enough endoscopic indicator to determine the management strategy of colorectal NENs. Our reports explored the value of the growth patterns of NENs in predicting pathological manifestations, chemotherapy sensitivity to first-line schemes and prognosis.

Numerous studies have focused on the macroscopic morphology of colorectal adenocarcinomas. Some reports classified tumors into polypoid and nonpolypoid types based on the presence or absence of elevated lesions compared with adjacent mucosa and concluded that nonpolypoid tumors exhibit more malignant characteristics and a poorer prognosis than polypoid tumors (19, 20). Others divided colorectal cancers into expansive, infiltrative and ulcerative subtypes or depressed, laterally spreading, protruding and ulcerative subtypes (13, 14). Unlike epithelial tumors, colorectal NEN is a rare subepithelial disease, and the classification system from adenocarcinomas may not be suited for NENs.

Several previous reports have explored the predictive value of the endoscopic features of NENs, but most of them focused on early, localized lesions less than 2 cm in size (21, 22). Normally, endoscopic evaluation for NENs includes an analysis of shape, color, and surface changes (depression, erosion, hemorrhage, ulceration and hyperemia) (23). Some studies have divided NENs into lesions with typical endoscopic features and lesions with atypical endoscopic features (24, 25). NENs with typical endoscopic findings appear as yellowish, sessile, smooth and submucosal tumors. They present favorable clinicopathological manifestations and clinical outcomes (26, 27). NENs with an atypical endoscopic appearance are morphologically unusual, showing irregular surfaces with depressions, ulceration, erosion, hemorrhage and hyperemia or being pedunculated. They are associated with high frequencies of lymph node metastasis, distant metastasis and a poor prognosis (23, 25). Moreover, some reports have indicated that NENs with a central depression on the surface had a higher tendency for incomplete endoscopic excision (28). Subsequently, Xiang-Yao Wang et al. classified NENs into type I (protruded), II (flat and slightly elevated) and III (depression and ulcer on the surface) and demonstrated that patients with type II and III NENs had a higher risk of incomplete endoscopic resection (21). In 2020, Luohai Chen et al. proposed a novel scoring system based on the endoscopic assessment of the size, shape and mucosal surface of primary rectal tumors, and it showed great value in identifying patients with endoscopically advanced disease and for monitoring tumor recurrence (22). However, most of the patients included in these reports had small, early and localized disease, and most NENs were indolent, diminutive, of a low grade and well differentiated. In addition, most of these previous studies focused only on the value of the endoscopic appearance of NENs in endoscopic therapy, and few have explored their value in predicting clinicopathologic features, chemotherapy sensitivity and clinical outcomes. Although locally advanced and metastatic NENs constitute only a small portion of colorectal NENs, they are usually characterized by greater malignancy, increased aggressiveness and a worse prognosis than localized NENs (29). Locally advanced and metastatic NENs therefore deserve more clinical attention, and our study focused on the value of cancer morphology in the management of these colorectal NENs.

We categorized stage II-IV colorectal NENs into protruding and ulcerative subtypes based on endoscopic evaluation of the presence or absence of elevated lesions compared with adjacent mucosa. The ulcerative group was characterized by significantly more malignant features than the protruding groups, including larger tumor sizes, higher frequencies of G3 NENs and poorly differentiated NECs, an increased Ki-67 index, and a higher proportion of T3 and T4 NENs. In the stratification analysis based on the presence or absence of distant metastasis, all these increased malignant characteristics were further verified for ulcerative NENs with regional disease. Regarding patients with metastatic diseases, size, grade and differentiation followed a similar path on ulcerative NENs group. In the stratification analysis based on tumor size, the increased Ki-67 index, higher frequencies of G3 NENs, NECs and T3 and T4 NENs of ulcerative group compared to protruding group were only statistically confirmed in patients with NENs size ≤ 2cm. For patients with NENs size > 2cm, no significant difference was observed, which may be due to the small sample size of our study. Overall, it may suggest that ulcerative NENs might be more aggressive than protruding NENs. For NENs ≤ 2cm, tumor macroscopic morphology may serve as an important reference index, NENs with ulcerative shape might not be suitable for endoscopic resection.

Given the rarity of colorectal NENs, there are no widely acknowledged optimal systematic chemotherapy regimens for the treatment of metastatic or recurrent disease. Most physicians adopt chemotherapy recommendations for colorectal adenocarcinomas and pulmonary NENs (30). In summary, temozolomide regimens (temozolomide plus capecitabine) and platinum regimens (cisplatin or carboplatin plus etoposide) are the cornerstones of first-line chemotherapy strategies for colorectal NETs and NECs, respectively. Moreover, the response rates vary widely between 14% and 75% according to the literature reports, and few markers have been found to predict the efficacy of systematic chemotherapy (10, 11). A total of 47 patients had evaluable data for first-line chemotherapy in our study, including 22 patients with protruding NENs and 25 with ulcerative NENs. Patients with protruding NENs had a significantly higher response rate than those with ulcerative NENs [50% (95% CI: 27.3% - 72.7%) versus 20% (95% CI: 3.1% - 36.9%), P=0.03], which implied that macroscopic morphology might be a valuable tool in predicting the chemotherapeutic sensitivity of colorectal NENs.

The 3-year PFS rates were 38.4% (95% CI: 29.2% - 47.6%), 49.5% (95% CI: 37.5% - 61.5%), and 19.7% (95% CI: 7.2% - 32.2%), and the 3-year OS rates were 57.2% (95% CI: 45.6% - 65.6%), 76.9% (95% CI: 66.5% - 87.3%), and 30.7% (95% CI: 15.6% - 45.8%) for the entire cohort, protruding NENs and ulcerative NENs, respectively. Patients with ulcerative NENs had significantly worse PFS and OS rates than protruding NENs, which was further confirmed in further univariable and multivariable Cox proportional hazards regression analyses after controlling for confounding factors. The 3-year cumulative incidence of CSM were 60.4% and 19.5% in ulcerative group and protruding group, respectively. This difference between the two groups were statistically confirmed through competitive risk analysis model. This result indicated that tumor shape might be a strong candidate for predicting the clinical outcomes of colorectal NENs. Patients with ulcerative NENs have a higher risk of tumor progression and cancer-specific death and require more intensive treatment and surveillance strategies than those with protruding NENs.

To our knowledge, macroscopic morphology has long been ignored in the current management of stage II-IV colorectal NENs, and few studies have explored its significance in predicting chemotherapy sensitivity, tumor recurrence and progression and survival outcomes. Our report demonstrated that gross morphology should be taken into account as an important parameter in the diagnosis, treatment and surveillance of these colorectal NENs. However, our study had the following limitations. First, due to the retrospective nature of our report, we enrolled patients over a period of 20 years, and bias from patient selection and data collection could not be completely avoided. Second, the sample size of our study cohort was relatively small; we included only 125 cases so our conclusions need to be confirmed in multicenter studies with larger sample sizes.

In conclusion, endoscopic evaluation of the macroscopic morphology of NENs may have a role in the management of stage II-IV colorectal NENs. In our cohort, ulcerative NENs present more malignant and aggressive potential, poor response to first-line chemotherapy regimens and decreased rates of PFS and OS when compared to protruding NENs. Tumor shape should be evaluated as an independent factor in the management of advanced colorectal NENs.

## Data Availability Statement

The raw data supporting the conclusions of this article will be made available by the authors, without undue reservation.

## Ethics Statement

The studies involving human participants were reviewed and approved by National Cancer Center/National Clinical Research Center for Cancer/Cancer Hospital, Chinese Academy of Medical Sciences. The patients/participants provided their written informed consent to participate in this study. Written informed consent was obtained from the individual(s) for the publication of any potentially identifiable images or data included in this article.

## Author Contributions

ZW anlyszed the data and wrote this article. KA and RL collected the data. QL designed this study and reviewed the manuscript. All authors contributed to the article and approved the submitted version.

## Funding

This study is supported by the following grants: the National Key Research and Development Program/Prevent and Control Research for Important Non-Communicable Diseases (No.2019YFC1315705), the Medicine and Health Technology Innovation Project of the Chinese Academy of Medical Sciences (No. 2017-12M-1-006), and the Special Fund of China Cancer Research Foundation/Beijing Hope Marathon (No. LC2017L03).

## Conflict of Interest

The authors declare that the research was conducted in the absence of any commercial or financial relationships that could be construed as a potential conflict of interest.

## Publisher’s Note

All claims expressed in this article are solely those of the authors and do not necessarily represent those of their affiliated organizations, or those of the publisher, the editors and the reviewers. Any product that may be evaluated in this article, or claim that may be made by its manufacturer, is not guaranteed or endorsed by the publisher.
